# Mesenchymal Stromal Cell-Produced Components of Extracellular Matrix Potentiate Multipotent Stem Cell Response to Differentiation Stimuli

**DOI:** 10.3389/fcell.2020.555378

**Published:** 2020-09-22

**Authors:** Ekaterina Novoseletskaya, Olga Grigorieva, Peter Nimiritsky, Nataliya Basalova, Roman Eremichev, Irina Milovskaya, Konstantin Kulebyakin, Maria Kulebyakina, Sergei Rodionov, Nikolai Omelyanenko, Anastasia Efimenko

**Affiliations:** ^1^Institute for Regenerative Medicine, Medical Research and Education Center, Lomonosov Moscow State University, Moscow, Russia; ^2^Faculty of Medicine, Lomonosov Moscow State University, Moscow, Russia; ^3^Faculty of Biology, Lomonosov Moscow State University, Moscow, Russia; ^4^N.N. Priorov National Medical Research Center of Traumatology and Orthopedics, Moscow, Russia

**Keywords:** extracellular matrix, decellularization, mesenchymal stromal cells, stem cell niche, progenitor

## Abstract

Extracellular matrix (ECM) provides both structural support and dynamic microenvironment for cells regulating their behavior and fate. As a critical component of stem cell niche ECM maintains stem cells and activates their proliferation and differentiation under specific stimuli. Mesenchymal stem/stromal cells (MSCs) regulate tissue-specific stem cell functions locating in their immediate microenvironment and producing various bioactive factors, including ECM components. We evaluated the ability of MSC-produced ECM to restore stem and progenitor cell microenvironment *in vitro* and analyzed the possible mechanisms of its effects. Human MSC cell sheets were decellularized by different agents (detergents, enzymes, and apoptosis inductors) to select the optimized combination (CHAPS and DNAse I) based on the conservation of decellularized ECM (dECM) structure and effectiveness of DNA removal. Prepared dECM was non-immunogenic, supported MSC proliferation and formation of larger colonies in colony-forming unit-assay. Decellularized ECM effectively promoted MSC trilineage differentiation (adipogenic, osteogenic, and chondrogenic) compared to plastic or plastic covered by selected ECM components (collagen, fibronectin, laminin). Interestingly, dECM produced by human fibroblasts could not enhance MSC differentiation like MSC-produced dECM, indicating cell-specific functionality of dECM. We demonstrated the significant integrin contribution in dECM-cell interaction by blocking the stimulatory effects of dECM with RGD peptide and suggested the involvement of key intracellular signaling pathways activation (pERK/ERK and pFAK/FAK axes, pYAP/YAP and beta-catenin) in the observed processes based on the results of inhibitory analysis. Taken together, we suppose that MSC-produced dECM may mimic stem cell niche components *in vitro* and maintain multipotent progenitor cells to insure their effective response to external differentiating stimuli upon activation. The obtained data provide more insights into the possible role of MSC-produced ECM in stem and progenitor cell regulation within their niches. Our results are also useful for the developing of dECM-based cell-free products for regenerative medicine.

## Introduction

The extracellular matrix (ECM) is a complex three-dimensional network of interlaced fibrillar proteins, proteoglycans, multiple matrix protein macromolecules, anchored growth factors, and other bioactive components (Gattazzo et al., 2014; Mouw et al., 2014; Theocharis et al., 2016). Extracellular matrix provides physical support for cells and modulates cell functional activity, proliferation, adhesion, migration, acquisition of a specialized phenotype and its maintenance (Gattazzo et al., 2014; Humphrey et al., 2014). Tissue-specific ECM appears as a result of the unique composition and topography and provides a distinctive cell microenvironment in various tissue compartments, including stem cell niches (Gattazzo et al., 2014; Ahmed and Ffrench-Constant, 2016; Chermnykh et al., 2018; Novoseletskaya et al., 2019). Different ECM components may support stem cells and regulate their fate as well as contribute to the malignization of normal cells (Iozzo and Gubbiotti, 2018). Composition of ECM produced by different cell types, the assembly of these macromolecules into a functional three-dimensional structure and its role in cell differentiation, tissue morphogenesis and physiological tissue remodeling were intensively studied for several decades (Lu et al., 2011; Bonnans et al., 2014; Leavitt et al., 2016). However, despite the currently accepted important contribution of ECM to the regulation of these processes, the mechanisms of complex effects exerted by ECM produced by specific cells are poorly understood.

It is well-known that mesenchymal stem/stromal cells (MSCs) play a key role in the processes of tissue repair and regeneration, mostly due to the production of soluble bioactive molecules, extracellular vesicles and ECM, which components are predominantly produced within MSC secretome (Kalinina et al., 2015; Keshtkar et al., 2018; Gomez-Salazar et al., 2020). They also include small subpopulation of stem and progenitor cells that can differentiate in adipogenic, osteogenic, chondrogenic, and some other lineages. Presumably, stromal cell subtypes presented in heterogeneous population of MSCs have regulatory functions that at least partially mediated by produced ECM. After an injury MSC-produced ECM creates a certain basis for the restoration of tissue structure, directing the migration of other cells to the damaged zone and stimulation of blood vessels and nerves growth (Rubina et al., 2009; Mauney et al., 2010; Ode et al., 2010; Lopatina et al., 2019a,b). Emerging ECM provides cell attachment and prevents their death as well as regulates the fate of stem and progenitor cells in regenerating tissue (Gattazzo et al., 2014).

One of the most promising approach to model ECM-based cell microenvironment is a decellularization of extracellular matrices provided bioactive and biocompatible materials consisting of a complex assembly of fibrillar proteins, matrix macromolecules and associated growth factors that often recapitulates the composition and organization of the original ECM microenvironments and creates the necessary microenvironment for the activity of cells *in vitro* or *in vivo* (Rana et al., 2017; Dzobo et al., 2019; Heath, 2019; Novoseletskaya et al., 2019; Ebrahimi Sadrabadi et al., 2020). To stimulate the production of ECM components MSCs can be cultured in 3D conditions such as cell multilayers, or cell sheets. Decellularization of cell sheets provides the preparation of ECM with a composition of protein components close to the native structure and composition (Cheng et al., 2014; Sart et al., 2020). Different decellularizing agents might be used including detergents, enzymes, apoptosis inductors, etc., and an effective combination should be adjusted based on required conservation of ECM structure and effectiveness of DNA removal (Gilpin and Yang, 2017; Hoshiba, 2017; Shakouri-Motlagh et al., 2017; Cramer and Badylak, 2019; Heath, 2019).

Several studies have shown the capacity of dECM to maintain the multipotent state of both MSCs (Lai et al., 2010) and hematopoietic stem cells (Prewitz et al., 2013). Concerning that ECM is one of the key components of the stem cell microenvironment that plays an important role in the regulation of stem cell self-maintenance and differentiation, dECM could be helpful for studying molecular interactions between stem/progenitor cells and ECM and establishing the role of cell-specific ECM in these processes. In the present study we developed the optimized preparation protocol of dECM produced by human MSCs with retained structure and composition. The obtained material was biocompatible, non-cytotoxic, non-immunogenic, and had the capacity to maintain cell growth. Then we evaluated the ability of dECM to restore stem and progenitor cell microenvironment *in vitro* and analyzed the possible mechanisms of its effects.

## Materials and Methods

### Cell Lines

ASC52telo, human telomerase reverse transcriptase (hTERT) immortalized adipose derived mesenchymal stem cells (ATCC^®^ SCRC-4000^TM^) (hTERT-MSCs) were received from ATCC^®^. hTERT-MSCs were used to assemble cell sheets and to obtain decellularized ECM (dECM). Primary MSC cell lines were isolated from subcutaneous adipose tissue human MSCs (hMSCs) and fibroblast cell lines were isolated from dermis (hFibroblast). All primary cell lines were obtained from healthy donors and preserved in the biobank of the Institute for Regenerative Medicine, Medical Research and Education Center, Lomonosov MSU, collection ID: MSU_MSC_AD, MSU_FB. The institutional local ethic committee (Ethic Committee of Lomonosov Moscow State University, IRB00010587) approved the collection of biomaterials from donors (protocol #4, date of approval 04.06.2018), and all donors provided the informed consent. Mesenchymal stem cells of all types were cultivated in AdvanceSTEM^TM^ media (HyClone, United States) supplemented with 10% Mesenchymal Stem Cell Growth AdvanceSupplement^TM^ (HyClone, United States), 1% penicillin/streptomycin solution (HyClone, United States), 1% GlutaMAX-1 (Gibco, United States). Dermal fibroblasts were cultivated in DMEM low glucose (Gibco, United States) supplemented with 10% fetal bovine serum (FBS; HyClone, United States), 1% penicillin/streptomycin solution (HyClone, United States), 1% GlutaMAX-1 (Gibco, United States). Cell culture conditions composed an atmosphere of 5% CO_2_ at 37°C. Culture medium was changed every 3 days.

Human promonocyte THP-1 cell line were cultivated in RPMI1640 media (Gibco, United States) supplemented with 10% FBS (Gibco, United States), 1% penicillin/streptomycin solution (HyClone, United States), 1% GlutaMAX-1 (Gibco, United States), 1% HEPES (Gibco, United States), 0.0001% β-mercaptoethanol (Sigma, United States) under an atmosphere of 5% CO_2_ at 37°C.

### Preparation of MSC-Produced dECM

hTERT-MSCs were seeded on tissue culture polystyrene (plastic) plates at a density of 50,000 cells per ml and were cultured for 2 weeks. To remove cellular components cell sheets were treated with either 0.5% 3-[(3-cholamidopropyl)dimethylammonio]-1-propanesulfonate (CHAPS) or 0.1% sodium deoxycholate dissolved in Phosphate-buffered saline (PBS), then washed 3–5 times with sterilized Hank’s Balanced Salt Solution (HBSS; Paneco, Russia), and incubated with DNase I (50 U/ml, SciStore, Russia) at 37°C for 30 min. To obtain higher efficiency of decellularization protocol, cell sheets were treated with 500 nM rotenone (Sigma, Germany) to induce apoptosis 24 h before use of detergents.

Apoptosis induction in hTERT-MSCs was evaluated by flow cytometry. Cell suspension was labeled with annexin V (Invitrogen, United States) and 7-Aminoactinomycin D (7-AAD) (BioLegend, United States) and analyzed using cytometer MoFlo (Dako Cytomation, United States). The number of live cells in hTERT-MSCs cell sheets after the incubation with rotenone was estimated by the amount of adenosine triphosphate (ATP) using the ATPlite 1 step kit (PerkinElmer, United States), according to the manufacturer’s instructions.

### Analysis of dECM Structure and Composition

Samples of hTERT-MSCs cell sheets and dECM were fixed in 10% neutral formalin, then they were washed in distilled water and dehydrated in alcohols of ascending concentration, a mixture of alcohol-acetone and then pure acetone. Samples were dried at the critical point using Quorum K350 (Quorum gala instrument gmbh, Germany) or HCP-2 (Hitachi, Japan) was used. The samples were mounted on a special aluminum table with conductive carbon glue, sprayed with gold or platinum-palladium alloy in the spraying unit Quorum Q150TS (Quorum gala instrument gmbh, Germany) or IB-3 Ion Coater (EIKO, Japan) and observed with scanning electron microscope S 3400N (Hitachi, Japan).

The structure of dECM was evaluated in Dulbecco’s Phosphate-Buffered Saline (DPBS) solution using a direct two-photon laser confocal scanning microscope A1rMP+ (Nikon, Japan) with NIR Apo 60x Oil λS DIC N2 lens. A titanium-sapphire laser Chameleon Vision II (Coherent, United States) with tunable wavelength in the range of 700–1000 nm, pulse repetition frequency of 80 MHz and pulse duration of 140 FS was used as a source of laser radiation. The excitation of the signal in this work was carried out at a wavelength of 760 nm. The signal was recorded simultaneously in three spectral channels: in the wavelength range 300 < λ1 < 400 nm (encoded in blue in the images), in the range 458 < λ2 < 492 nm (encoded in green), and in the range 492 < λ3 < 593 nm (encoded in yellow). Processing of the images was performed in the program NIS-Elements (Nikon, Japan).

Type I collagen, fibronectin and laminin in the deposited ECM were investigated by immunohistochemical labeling. First, the deposited ECM after decellularization were detached from the plastic and placed in Tissue-Tek^®^ O.C.T. Compound (Sakura^®^ Finetek, United States) freezing medium. After sample polymerization the 8 μm cryosections were prepared using Leica CM1950 cryostat (Germany). The sections were fixed with 4% paraformaldehyde (Panreac, Spain) at room temperature for 30 min and then samples were incubated with 0.2% triton X100 (Sigma, United States) solution at room temperature for 10 min. Further, the samples were incubated with 1% bovine serum albumin (BSA, Sigma, United States) and 10% normal goat serum (Abcam, United Kingdom) solution at room temperature for 1 h. Subsequently, the samples were incubated with rabbit polyclonal primary antibodies: anti-collagen I (ab34710), anti-fibronectin (ab2413) and anti-laminin (ab11575) (Abcam, United Kingdom) in 1% BSA solution at +4° overnight. Samples were then incubated with goat-anti-rabbit secondary antibodies (A11034, A11037, Invitrogen, United States) at room temperature for 1 h. Cell nuclei were labeled with DAPI (DAKO, United States). Another approach included labeling ECM components of cell sheets or dECM attached to plastic. Samples were fixed with 4% paraformaldehyde (Panreac, Spain) at room temperature for 10 min and were processed as described above. hMSCs seeded on plastic or dECM were treated as described above using anti-β1-antibody (303001, Biolegend, United States). Samples were analyzed with a Leica DM6000B fluorescent microscope equipped with a Leica DFC 360FX camera (Leica Microsystems GmbH, Germany).

### Evaluation of Residual DNA Content

The amount of DNA in hTERT-MSCs cell sheets or dECM was measure using PicoGreen assay kit (Life Technologies, United States) according to the manufacturer’s instructions. Each sample was lysed in 500 ml of ExtractRNA (Eurogen, Russia) (analogous reagent as TRIzol, TRI Reagent) for 10 min at room temperature. The lysates were diluted 50 times with TE buffer solution. Dissolved lysate was placed in a 96-well plate with PicoGreen dye (1:1). The plate was incubated in the dark for 10 min and then the optical density was measured with a plate spectrophotometer EnVision Multilabel Plate Readers (PerkinElmer, United States).

### *In vitro* Time-Lapse Evaluation of MSC Proliferation With IncuCyte ZOOM System

hTERT-MSCs were seeded in a 12-wells plate, covered with dECM (15,000 cells per ml). The plate was placed in the IncuCyte^®^ ZOOM Live Cell Analysis System (Essen Bioscience, United States) and time-lapse shooting was carried out every 4 h for 4 days. The device’s built-in software allows to estimate the area occupied by cells by applying a “mask” to the obtained images and thus calculating the percentage of cell culture confluency. The increase in confluence directly correlates with the increase in the number of cells, which allows us to judge the growth rate of cell culture by calculated parameters.

To analyze the proliferation rate of hTERT-MSCs, cells were seeded on culture plastic or dECM and cultured for 4 days. Cell number was examined by MTT test (Paneco, Russia) on day 1 and 4. Optical density was measured with the plate spectrophotometer EnVision Multilabel Plate Reader (Perkin Elmer, United States).

### Immunogenic Properties of dECM

Immunogenic examination was evaluated through the ability of dECM to induce macrophage differentiation. When tissue is injured, monocytes appear in the damaged area after a few hours, where they begin to differentiate into macrophages. Thus, during the entire healing process, macrophages are one of the main regulators of repair, with a powerful paracrine effect. A distinctive feature of the line of monocytes-macrophages is plasticity, which turns them into an adaptive component of innate immunity (Locati et al., 2013). *In vitro* monocyte activation test (MAT) is widely used to assess the pyrogenic activity of medical materials and detect substances that activate human monocytes to release cytokines (Borton and Coleman, 2018). THP-1 is a human leukemia monocytic cell line, which has been extensively used to study monocyte/macrophage functions, mechanisms, signaling pathways, and nutrient and drug transport. This cell line has become a common model to estimate modulation of monocyte and macrophage activities (Chanput et al., 2014). THP-1 are regarded as a simplified model of human macrophages when investigating relatively straightforward biological processes, such as polarization and its functional implications (Tedesco et al., 2018). To perform MAT for investigating immunogenic properties of prepared dECM, THP-1 were added to a 12-well plate on plastic or dECM (500,000 cells per well) with or without 50 ng/ml phorbol ester (phorbol-12-myristate-13-acetate, PMA) (Sigma, United States). Cells were cultured for 7 days, then conditioned medium was collected and centrifuged at 300 *g* for 10 min. The concentration of IL-6, IL-8, IL-10, IL-12p70, MCP-1 was analyzed in supernatants using enzyme-linked immunoassay (ELISA; R&D, United States) according to the manufacturer’s instructions. The optical density of the samples was measured at wavelengths of 450 and 525 nm with a flatbed spectrophotometer EnVision Multilabel Plate Reader (PerkinElmer, United States).

### Colony-Forming Unit Assay

To determine the ability of dECM to support MSC colony formation, colony-forming unit (CFU) assay was performed. MSCs were seeded on plastic or dECM at low density (400 cells per well in 6-well culture plate) and cultured for 2 weeks, then fixed with a solution of 4% paraformaldehyde and stained with crystal violet (Sigma, United States) for 30 min at room temperature. The nuclei we additionally stained with DAPI. The number and average size of colonies as well as area of cell growth and cell number were counted in each well.

### Adipogenic, Osteogenic, and Chondrogenic Differentiation of MSCs

Cell differentiation was performed using the StemPro Adipogenesis Differentiation Kit (Gibco, United States), StemPro Osteogenesis Differentiation Kit (Gibco, United States) and StemPro Chondrogenesis Differentiation Kit (Gibco, United States) according to the manufacturer’s instructions. Briefly, hMSCs were seeded in 12-well plates in the density of 120,000 cells per well (and in 48-well plates in the density of 30,000 cells per well) on dECM produced by hTERT-MSCs or dermal fibroblasts, on plastic or on plastic covered by ECM structural proteins [recombinant human fibronectin or recombinant human laminin or type I collagen (IMTEK, Russia)] according to the manufacturer’s recommendation for cell culture. Then cells were and cultured for 24 h in the full growth media. After 24 h the media has been replaced by differentiation induction media. The media was changed every 3 days. Four or 10 days after induction samples were fixed with 4% paraformaldehyde for 30 min. Visualization of mineral deposits by osteoblasts was performed with Alizarin red solution from Mesenchymal Stem Cell Osteogenesis Kit (Chemicon, United States), lipid drops in adipocytes were stained with Oil Red O solution from Mesenchymal Stem Cell Adipogenesis Kit (Chemicon, United States) according to the manufacturer’s instructions. Chondrogenic differentiation was observed with staining 0.1% Toluidine blue (Sigma, United States) solution in 1% Sodium chloride, pH 2.3. For quantification, all of dyes were extracted with DMSO (AplliChem, United States), and the absorbance at 530 nm (Oil Red O), at 560 (Alizarin Red) and at 608 nm (Toluidine blue) was measured on a flatbed spectrophotometer EnVision Multilabel Plate Reader (PerkinElmer, United States).

### Real-Time RT-PCR

Total RNA was extracted from 120 × 10^3^ cells (∼90% confluence) using the RNeasy Mini Kit (Qiagen, United Kingdom) following the manufacturer’s protocols. The cDNA was synthesized with 300 ng of total RNA using the MMLV Reverse Transcription Kit (Eurogen, Russia). The temperature mode was supported by Mastercycler^®^ nexus gradient (Eppendorf, Germany). cDNA was used for qPCR with qPCRmix-HS SYBR + LowROX (Eurogen, Russia) according to the manufacturer’s protocols. Gene expression analysis was performed with the relative quantification method. Quantification and normalization of expression levels of the target genes and the reference gene (RPLP0) were calculated using the comparative threshold cycle (CT) method. RPLP0 is one of the most stable expression housekeeping gene during hMSCs differentiation into the canonical lineages (Ragni et al., 2013). The primers were designed in-house using the NCBI Primer Designing Tool,^1^ selecting only the primers spanning an exon–exon junction and producing a PCR amplificate with length between 63 and 199 base pairs (Supplementary Table 1).

Real-time PCR data were analyzed using the ΔC_*T*_ method for evaluating the expression of master genes and markers of adipogenic and osteogenic differentiation normalized to housekeeping gene (RPLP0) in dynamics, as well as analysis of pluripotency gene expression and integrin expression profile. We applied the 2^–ΔΔ*CT*^ method to evaluate the level of target gene expression in experimental samples relative to untreated control samples (without differentiation induction) in integrin-blocking assays.

### Inhibitor Analysis and Integrin-Blocking Assays

hMSCs were seeded in 48-well plates in the density of 30,000 cells per well for differentiation experiment and in six-well plates in the density of 300,000 per well for western blot assay, on plastic or on dECM produced by hTERT-MSCs. Then cells were cultured for 24 h in the full growth media. After 24 h the media has been replaced by media, consist of growth media with one of the next signaling inhibitors: inhibitor of the Src family of protein tyrosine kinases (PP2) (529573, Sigma, United States), PD 98059 selective & reversible inhibitor of MAP Kinase Kinase (MEK; 513000, Sigma, United States), Akt Inhibitor VIII, Akti-1/2 (Akti) (124018, Sigma, United States) and dobutamine hydrochloride (DBN) as inhibitor of YAP nuclear translocation (DBN) (ab120768) (Abcam, United Kingdom) (Bao et al., 2011). All inhibitors were added to the final concentration of 10 mkM (Kim and Kim, 2010; Hu et al., 2014; Shin et al., 2019), except for DBN up to 20 mkM (Lorthongpanich et al., 2019). Therefore, 0.1% DMSO was used as a vehicle control in all experiments. After another 24 h, signaling inhibition was evaluated using Western blot. In another case, the medium was changed to induction medium for inhibition of signaling during differentiation of cell for 4 days. On the 4th day, the cells were fixed and the studied as described in section “Adipogenic, Osteogenic, and Chondrogenic Differentiation of MSCs.”

hMSCs were incubated with cycle-RGD-TPP peptide (kindly provided by Elena Markvicheva, Institute of bioorganic chemistry of RAS, Moscow, Russian Federation), which blocked the connection of integrins with ECM proteins consisted RGD sequence. Ñycle-RGD-TPP was used at the empirically adjusted concentration of 40 μM. Cell suspension were incubated during 10 min under cell culture conditions in solution of Ñycle-RGD-TPP. hMSCs were then seeded onto surfaces in the density of 120,000 cells per well in culture media or differentiation media, while peptide still present and not allowed to attach for 4 days.

### Western Blotting and Dot-Blotting

Proteins were extracted using cell lysis buffer [62.5 mM Tris-HCl (pH 6.8), 7.5% glycerol, 2% SDS, 0.0125% Bromophenol blue and 1.25% β-mercaptoethanol] supplemented with a protease inhibitor cocktail (Roche, South San Francisco, CA, United States) and HaltTM phosphatase inhibitor cocktails (Thermo Fisher Scientific, United States). Nuclear and cytoplasmic extracts were obtained as described by Longobardi and Blasi (2003). About 500 × 10^3^ cells (∼100% confluence) were washed twice with cold phosphate-buffered saline, separated by centrifugation (200 *g* 5 min + 4°C), collected with 150 μl of cold buffer A (10 mM HEPES, pH 7.9, 10 mM KCl, 1.5 mM MgCl_2_, 0.5 mM dithiothreitol, 0.5 mM phenylmethylsulfonyl fluoride) into an Eppendorf tube, left 10 min on ice, and lysed by the addition of 0.3% Triton X-100. The nuclei were collected by centrifugation (14,000 *g* 5 min + 4°C). The supernatant was transferred to a new tube, and 0.11 volume of buffer B was added (0.3 M HEPES, pH 7.9, 1.4 M KCl, 30 mM MgCl_2_), incubated for 30 min at 4°C, and centrifuged (14,000 *g* 5 min + 4°C). The resulting supernatant was denoted as cytoplasmic extract. Nuclear extracts were prepared by resuspending the pelleted nuclei in 30 μL of buffer C (20 mM HEPES, pH 7.9, 25% glycerol (v/v), 0.42 M NaCl, 1.5 mM MgCl_2_, 0.5 mM dithiothreitol, 0.2 mM EDTA, 0.5 mM phenylmethylsulfonyl fluoride) for 30 min on ice. The extract was cleared by centrifugation (14,000 *g* 5 min + 4°C). Protein concentration was determined using a Bradford protein assay.

Polyacrylamide gel electrophoresis was performed according to the standard protocol (Laemmli, 1970). After electrophoretic separation by 12% SDS-polyacrylamide gel electrophoresis, proteins were electroblotted onto polyvinylidene fluoride (PVDF) membranes (Millipore Immobilon-P) according to the method described by Towbin et al. (1979), or the gel was stained with a Coomassie Blue Silver suspension as described in Candiano et al. (2004). The membranes were blocked with Tris-buffered saline containing 0.1% Tween-20 (TBST) and 5% BSA for 30 min at room temperature. For target protein detection following primary antibodies were used: anti-collagen I (ab34710), anti-fibronectin (ab2413), anti-laminin (ab11575) (Abcam, United Kingdom), Anti-ERK1 + ERK2 antibody (Abcam; ab17942), Anti-Erk1 (pT202/pY204) + Erk2 (pT185/pY187) antibody (Abcam, ab4819), Anti-Active-β-Catenin (Anti-ABC) (Merck Millipore, 05-665), Anti-β-Catenin Antibody (Merck Millipore, 6734), YAP Antibody (Cell signaling technology, 4912), Phospho-YAP (Ser127) Antibody (Cell signaling technology, 4911), Phospho-FAK (Tyr397) Antibody (Cell signaling technology, 3283), FAK Antibody (Cell signaling technology, 3285), Phospho-Akt (Ser473) Antibody (Cell signaling technology, 9271), Akt (pan) (C67E7) Antibody (Cell signaling technology, 4691), p-c-Src (Tyr 419)(Santa Cruz Biotechnology, sc-101802), Anti-Vinculin antibody (Merck Millipore, V4139), histone H3 (Cell Signaling Technology, 4499) for nuclear fraction and GAPDH for total and cytoplasmic fractions (14C10) (Cell Signaling Technology, 2118). Membranes were incubated with antibodies overnight at 4°C with slight shaking. Thereafter, the membranes were washed in TBST buffer and further incubated with an HRP-conjugated antibody (goat anti-rabbit IgG; IMTEK) for 2 h at room temperature. To visualize the protein bands an enhanced chemiluminescence system (Bio-Rad ECL Clarity Max detection kits with the ChemiDoc Imaging System Bio-Rad, United States) was used. Semi-quantitative evaluation of the bands was performed by densitometry. The relative levels of FAK, p-FAK, ERK1/2, p-ERK1/2, YAP, and pYAP were determined by normalizing them to the level of the housekeeping protein GAPDH, the relative level of active b-catenin in nuclear fraction was normalized to histone H3 protein.

Quantitative analysis of proteins of interest (collagen-1, fibronectin, laminin) was carried out by the method of dot-immunoenzyme analysis (dot-ELISA): lysed samples were dispensed and dried per 1 μl in two parallels on nitrocellulose membrane surface. Then immunodetection by the above protocol for Western blotting was carried out. The proteins of interest were visualized using the Clarity Max ECL chemoluminescence kit (Bio-Rad, United States). In addition, in parallel with the lysate samples, samples with a known concentration of a specific protein of interest were titrated with 1: 2 dilution factor and processed in the same way to construct a calibration curve. Quantitative calculations were performed using ImageJ application (NIH, Bethesda, MD, United States). Briefly, measured chemiluminescence levels were used to calculate the concentrations of the proteins of interest using a calibration curves, and then the calculated amounts were normalized on total protein amounts in corresponding samples measured using Amido Black dye (Sigma Aldrich, United States).

### Statistical Data Processing

Statistical data processing was performed using the program GraphPad Prizm 8.0. Values are expressed as mean ± SD for normally distributed data and as median and percentiles (25–75%) for non-normal data. If normality of data were confirmed (according to the Kolmogorov–Smirnov test and Shapiro–Wilk’s W test), comparison of independent groups was performed by Student’s *t*-test, *F*-test, Tukey’s multiple comparisons test; if the data were not confirmed comparison was performed by Mann–Whitney test, Dunn’s multiple comparisons test, two-stage linear step-up procedure of Benjamini, Krieger and Yekutieli. Multiple comparisons were made using one-way ANOVA for normally distributed data and otherwise by Kruskall–Wallis test. Differences were considered statistically significant at the significance level of *p* < 0.05. Significant differences marked by ^∗^(*p* value < 0.05), ^∗∗^(*p* value < 0.005), ^∗∗∗^(*p* value < 0.0005) and ^****^(*p* value < 0.0001).

## Results

### Preparation of MSC-Produced dECM

**FIGURE 1:**
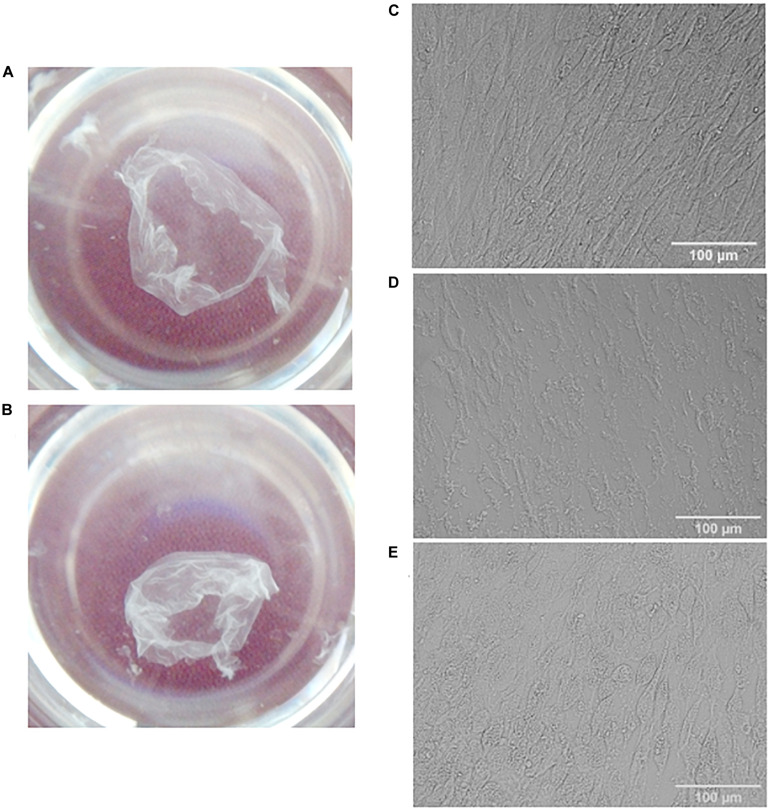
Visualization of MSC cell sheets **(A,C)** and decellularized cell sheets **(B,D,E)**. General view **(A,B)** and representative microphotographs of a cell sheet **(C)**, dECM prepared with sodium deoxycholate and DNAse I treatment **(D)** or with additional pre-incubation with rotenone **(E)**. Pictures were obtained using differential interference contrast (DIC) microscopy (objective magnification, ×10).

To promote ECM production, we cultured hTERT-MSCs as cell sheets for two weeks. Cells produced and formed ECM proteins (Figures 1A,C). Based on our previous results and literature data the following detergents for decellularization were selected: zwitter-ion detergent – CHAPS and ion detergent – sodium deoxycholate. Ionic detergents, such as sodium deoxycholate, are actively use for the decellularization of organs and tissues (Rana et al., 2017; Dzobo et al., 2019; Heath, 2019; Ebrahimi Sadrabadi et al., 2020; Sart et al., 2020). On the other side, this type of detergents can contribute the leaching of a significant part of the ECM from the cell sheet. Despite the properties of ionic detergents, as example sodium deoxycholate is FDA approved for medical use. Another perspective type of detergents for the decellularization of tissue and organs is zwitter-ionic surfactants (CHAPS). These surfactants also effective remove cell compartment, at the same time they do not denature proteins (Tsuchiya et al., 2014, 2016; Sengyoku et al., 2018), which makes them the most promising agent for the decellularization of cell sheets.

In addition, we hypothesized that pre-incubation of hTERT-MSCs cell sheets with rotenone (apoptotic inductor) may increase the efficiency of decellularization. As a promising approach for decellularization we examined one of the inductors of apoptosis (rotenone). Rotenone is a strong inhibitor of the mitochondrial complex I, which disrupt electron transfer within the mitochondrial respiratory chain leads to the formation of reactive oxygen species and thereby induces oxidative stress and apoptosis in cells. After detergent treatment and several washings cell sheets were incubated with DNase I in the empirically adjusted concentration (50 U/ml) selected from the tested interval 50–200 U/ml. Examples of obtained dECM are shown in Figure 1B,D,E.

The following combinations of decellularization agents were tested: CHAPS with DNAse I, rotenone with CHAPS and DNAse I, sodium deoxycholate with DNAse I, rotenone with sodium deoxycholate and DNAse I (Figure 1). Rotenone effectively induced apoptosis both in hTERT-MSCs suspension and hTERT-MSCs cell sheets (Supplementary Figure S1), although we did not find any improvement in decellularization efficiency based on residual DNA in dECM after the pretreatment with rotenone.

**FIGURE 2:**
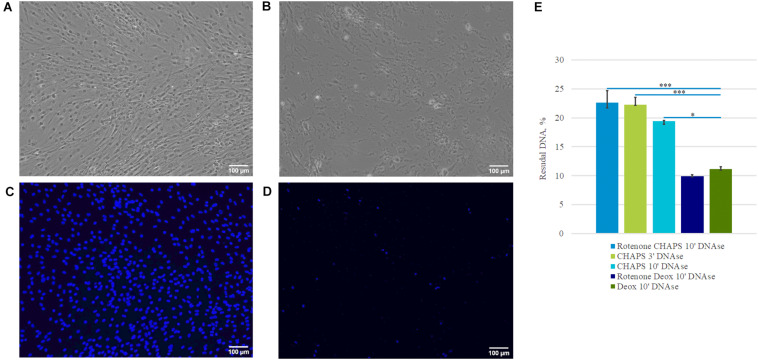
Effectiveness of decellularization accessed by the percentage of residual DNA. Representative microphotographs of a cell sheet **(A,C)**, dECM prepared with CHAPS and DNAse I treatment **(B,D)**. Pictures were obtained using phase-contrast **(A,B)** and fluorescence microscopy **(C,D)** – (blue is DAPI) (objective magnification, ×10). Examination of DNA content in dECM prepared by different methods was performed using PicoGreen assay and normalized to DNA content in the cell sheets **(E)**. The quantitative data are represented as median (25%, 75%), ^∗^(*p* value < 0.05), ^∗∗∗^(*p* value < 0.0005).

**FIGURE 3:**
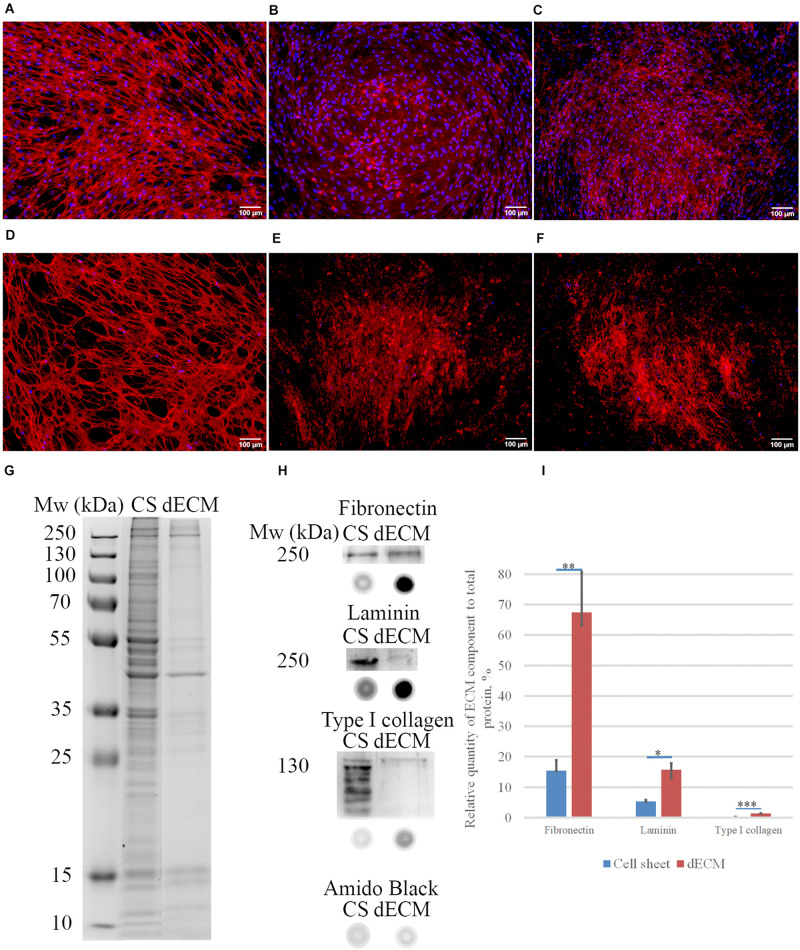
Expression of ECM proteins [fibronectin **(A,D)**, laminin **(B,E)**, type I collagen **(C,F)**] in MSC cell sheets and dECM evaluated by immunohistochemistry without permeabilization (objective magnification, ×10). Control immunostaining with isotype IgG is presented in Supplementary Figure S4. Silverstain analysis of electrophoretically separated proteins in cell sheet (CS) and dECM **(G)**. Immunoblot analysis of total fibronectin, total laminin and type I collagen expression levels in CS and dECM **(H)**. Also, dot-blot results performed **(H)**, and data analysis of quantity expression is shown **(I)**. The quantitative data are represented as median (25%, 75%), *(*p* value < 0.05), *^∗^(*p* value < 0.005), ^∗∗∗^(*p* value < 0.0005).

Decellularization efficiency was estimated by residual DNA as a percentage of the original DNA content in the cell sheets (Figure 2). Execution of all tested protocols effectively removed DNA (80 ± 5% efficiency). At the same time, using sodium deoxycholate led to better removal efficiency (Figure 2E), but the amount of residual matrix in this biomaterial was lower and its structure was substantially damaged compared to CHAPS-based protocols (Figure 1D and Supplementary Figure S3). Unexpectedly, there were no benefits of the protocols included incubation with rotenone concerning the decellularization efficiency (Supplementary Figures S2, 3). In all dECM samples we detected basic ECM structural proteins such as type I collagen and fibronectin via IHC analysis (Supplementary Figure S2). Another ECM protein laminin was found in low quantity. Better results were obtained when the treatment of CHAPS and DNAse I was used for decellularization: structural ECM proteins and their three-dimensional patterns were preserved which was confirmed by immunohistochemical analysis (Figure 3).

Biochemical assessment of matrix structure demonstrated that the total amount of protein decreased during decellularization (Figure 3G), but as a result of the procedure, the samples were enriched with ECM components (Figures 3H,I). The most represented component of ECM was fibronectin, and laminin was the second most represented component. Type I collagen was obviously not the major type of collagen produced by these cells in this approach, but its amount also increased relative to the total protein after decellularization.

**FIGURE 4:**
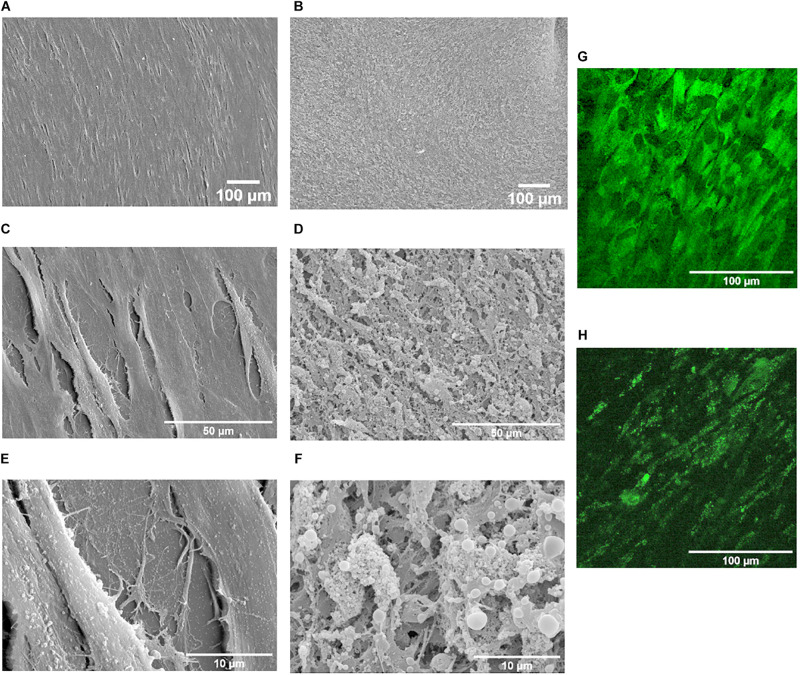
Scanning electron microscopy of MSC cell sheets **(A,C,E)** and dECM prepared by the treatment with CHAPS and DNAse I **(B,D,F)**. Magnification: ×100 **(A,B)**, ×1000 **(C,D)**, ×4000 **(E,F)**. Study of second harmonic generation (SHG): **(G)** MSC cell sheet, **(H)** dECM prepared by the treatment with CHAPS and DNAse I.

The microstructure of dECM was also studied using scanning electron microscopy (SEM) and two-photon laser microscopy. Decellularization of hTERT-MSCs cell sheets with sodium deoxycholate led to the destruction of protein network and mesh-like structure of ECM (Figure 1D and Supplementary Figures S3D,I). In contrast, preincubation with CHAPS for 10 min resulted to the better preservation of ECM (Supplementary Figures S2B,G), whereas a shorter preincubation for 3 min had kept the efficiency of decellularization (Figures 4B,D,F) and allowed to get a tight dimensional array with a scattered ECM cell debris and matrix-bounded vesicles (Figures 4D,F).

Two-photon laser microscopy allows to find out whether fibrillar proteins such as type I and II collagens are present in ECM. However, comparison of cell sheets and dECM using two-photon laser microscopy did not reveal the presence of any fibrillar proteins nor in hTERT-MSCs cell sheets, neither in dECM (Figures 4G,H).

**FIGURE 5:**
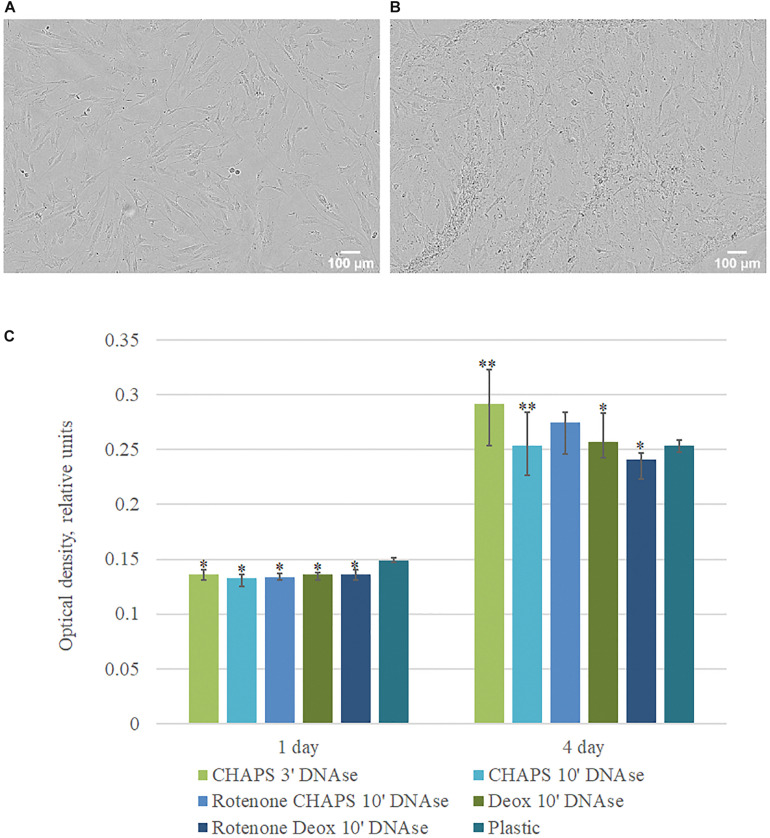
Proliferation activity of hMSCs cultured on plastic and dECM. **(A,B)** Representative microphotographs of hMSCs cultured on plastic **(A)** and dECM **(B)** for 4 days (phase contrast, objective magnification – ×10), **(C)** MTT assay of hMSCs cultured on plastic and dECM for 1 and 4 days. The quantitative data are represented as median (25%, 75%). Significant differences marked by ^∗^(*p* value < 0.05), ^∗∗^(*p* value < 0.005) compared to cells cultured on plastic.

To evaluate the proliferation and adhesion rate of hTERT-MSCs seeded on dECM, cells were placed in the Incucyte ZOOM system. Time-lapsed shooting showed that hTERT-MSCs adhered to dECM surface and proliferated during the entire period of the experiment. MTT test was performed to quantify hTERT-MSCs proliferation rate. We found significantly higher hTERT-MSCs proliferation activity if cultured on dECM obtained with CHAPS + DNAse I treatment comparing to other protocols (Figure 5). Taken together, prepared hTERT-MSCs-produced dECM is a biocompatible and non-cytotoxic material, which can maintain cell survival and proliferation. The protocol included the treatment with 0.5% CHAPS for 3 min with the following incubation with DNAse I was selected as the preferable decellularization method for MSC cell sheets and then was used in further research.

### dECM Modulate the Differentiation of THP-1 to Macrophages

Since none of the existing methods of decellularization provides complete DNA removal from the sample, there is an opportunity of potential immunogenicity of the residual DNA *in vivo*.

To test immunogenic properties of dECM, we used the monocytes to macrophages differentiation *in vitro* model. As monocytes we used THP-1 cell line which represents human promonocytes cells. We cultured THP-1 on plastic or dECM during a week with or without 50 ng/ml PMA. To evaluate the macrophage activation, the levels of pro-inflammatory and anti-inflammatory cytokines in the cell-conditioned medium were analyzed using ELISA for the determination of IL-6, IL-8, IL-10, IL-12, and MCP-1.

Phorbol-12-myristate-13-acetate stimulation led to the activation of THP-1, attachment of cell suspension to plastic/dECM surface and spreading which are typical signs of macrophage differentiation. Respectively, we observed the increased level of pro-inflammatory cytokines in conditioned media such as IL-6 (465.7 pkg/ml), MCP-1 (7.6 ng/ml), and IL-8 (1.5 μg/ml). We observed low levels of secreted IL-10 which correlates with classical pro-inflammatory macrophage polarization.

**FIGURE 6:**
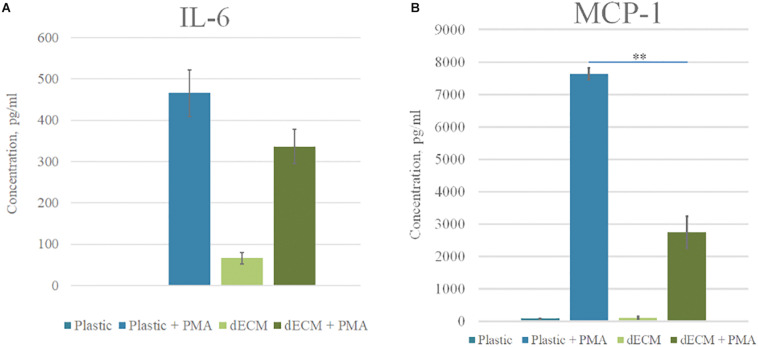
Cytokine profile of monocytes cultured on plastic and dECM. Level of IL-6 **(A)** and MCP-1 **(B)** secreted by monocytes/macrophages with or without PMA treatment measured by ELISA are presented. The quantitative data are represented as median (25%, 75%), ^∗∗^(*p* value < 0.005).

Decellularized ECM itself did not stimulate THP-1 activation and differentiation as followed from cellular morphology and low levels of secreted factors [IL-6 (6 pkg/ml), IL-8 (242.7–452.3 pkg/ml), MCP-1 (85–112 pkg/ml)], IL-10 (no secretion was observed), and IL-12 (no secretion was observed). At the same time, macrophages cultured on dECM with PMA secreted 2.8-times less MCP-1 and 1.4-times less IL-6 than macrophages cultured on plastic in the presence of PMA (Figure 6).

Thus, the stimulation of THP-1 by PMA resulted in differentiation toward classically activated macrophages M1. The investigated dECM did not stimulate macrophage differentiation and even reduced the concentration of pro-inflammatory factors in conditioned media. We concluded the absence of immunogenicity of dECM in our model. The decrease of IL-6 and MCP-1 secretion during cultivation of macrophages on dECM may consider as an anti-inflammatory effect, although this statement requires further investigations.

### Evaluation of dECM Influence on the Colony Formation and Stemness Maintenance

**FIGURE 7:**
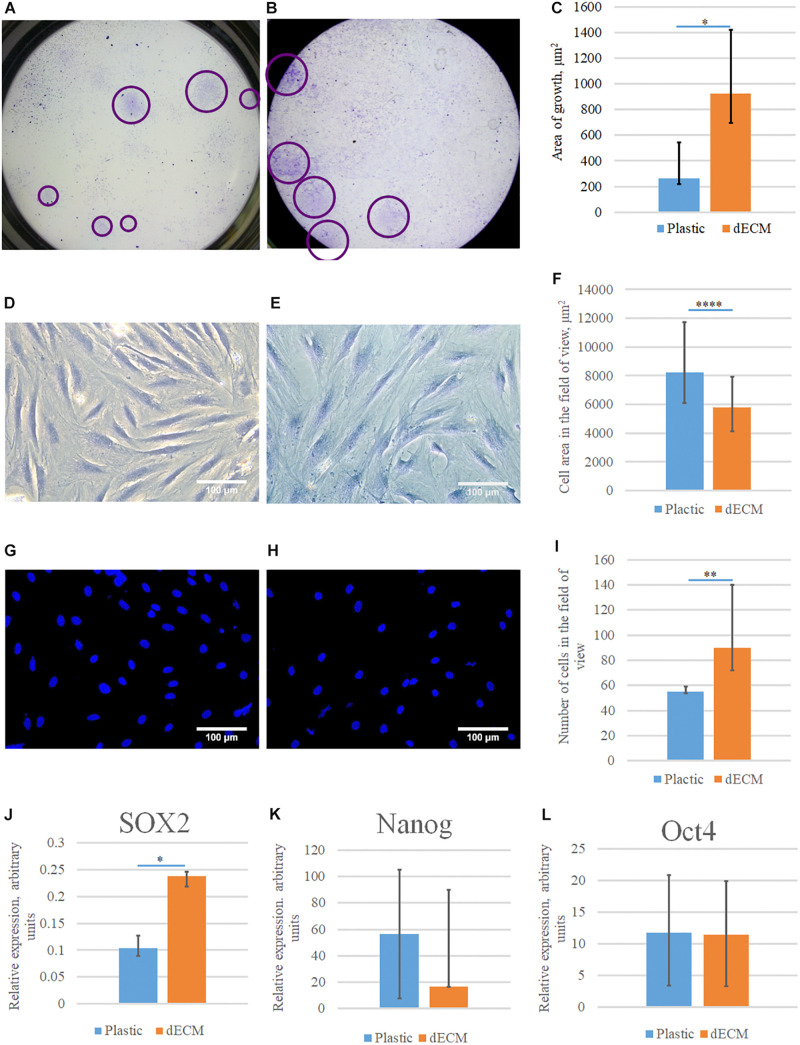
Evaluation of the colony formation and stemness maintenance of hMSCs cultured on plastic or dECM. CFU-F assay was performed for hMSCs cultured on plastic **(A,D,G)** or dECM **(B,E,H)**, quantification of differences between cell colony sizes, cell area in field of view or number of cells in the field of view are demonstrated on diagrams **(C,F,I)**. Relative expression of pluripotency genes *SOX2* **(J)**, *NANOG* **(K)**, and *POU5F1* (Oct4) **(L)** in hMSCs measured by real-time PCR normalized to housekeeping gene (RPLP0). The quantitative data are represented as median (25%, 75%), ^∗^(*p* value < 0.05), ^∗∗^(*p* value < 0.005), ^*⁣*⁣**^(*p* value < 0.0001).

We tested the ability of dECM to stimulate the formation of colonies by multipotent hMSCs comparing to cultural plastic in CFU assay. hMSCs were seeded on plastic or dECM and after 10 days of cultivation we evaluated the number of formed colonies. CFU assay showed that the number of clonogenic precursors were comparable in both cases (we observed 3% CFU on plastic vs 2.5% CFU on dECM), although it should be noted that the size of a single colony was significantly larger if hMSCs were cultured on dECM (Figures 7A,B,C). To exclude the influence of cell adhesion, a thorough analysis of cell morphology (Figures 7D–I) was performed, and it was shown that despite the larger area of colonies on the dECM, the area of each cell individually did not increase (Figure 7F). On the contrary, the area of cells on plastic was statistically significantly larger, although the number of cells was smaller (Figures 7F,I). It is likely that the native matrix formed by the cells themselves provides a division-friendly geometric and functional substrate for cell growth. Analysis of mRNA level of pluripotency genes (*NANOG, OCT4, SOX2*) in hMSCs did not show any substantial differences between plastic and dECM, however, *SOX2* expression was slightly increased in hMSCs cultured on dECM (Figures 7J,K,L). Thus, hTERT-MSCs-produced dECM does not significantly affect the number of stem cells and expression of pluripotency genes supporting “stemness” of cultured cells. However, it probably promotes active proliferation even at the level of a single progenitor cell.

### dECM Stimulates Inducible Differentiation of Primary MSCs

**FIGURE 8:**
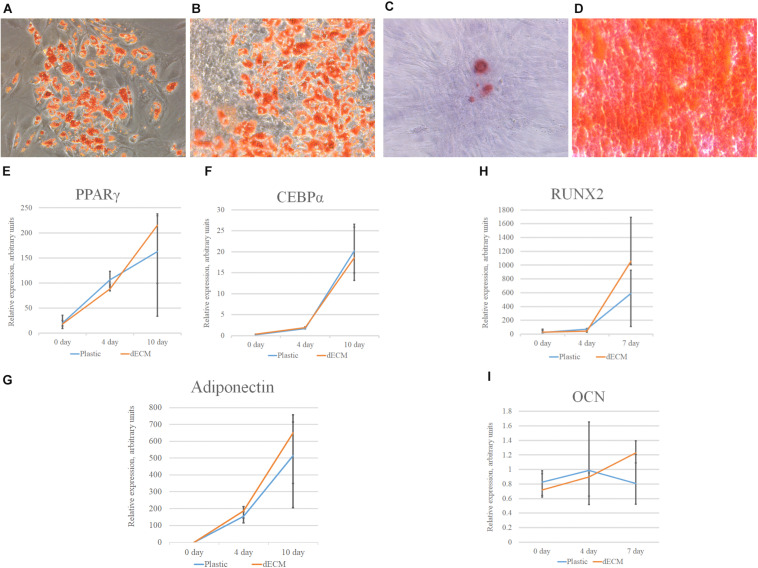
Induction of adipogenic and osteogenic differentiation of hMSCs cultured on plastic **(A,C)** or dECM **(B,D)** for 10 days assessed by cytochemical staining with Oil Red **(A,B)** or Alizarin Red **(C,D)** and expression of adipogenic [PPARγ(*PPARG*) **(E)**, CEBPα(*CEBPA*) **(F)**, adiponectin (*ADIPOQ*) **(G)**] or osteogenic [*RUNX2* **(H)**, osteocalcin(*OCN*) **(I)**] differentiation markers measured by real-time PCR normalized to housekeeping gene (RPLP0). Objective magnification, ×10. The quantitative data are represented as median (25%, 75%).

**FIGURE 9:**
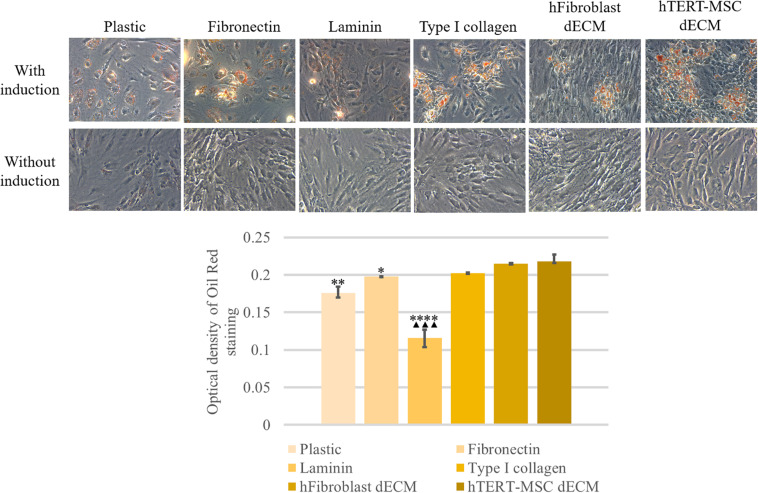
Induction of adipogenic differentiation of hMSCs cultured on plastic, plastic covered by fibronectin, or laminin, or type I collagen and dECM produced by human fibroblasts or MSCs for 4 days assessed by histological staining with Oil Red. Objective magnification, ×20. The dye was extracted, and Oil Red O staining was quantified by measuring the optical density at 530 nm **(lower panel)** (*n* = 4). The quantitative data are represented as median (25%, 75%), ^∗^(*p* value < 0.05), ^∗∗^(*p* value < 0.005), ^*⁣*⁣**^(*p* value < 0.0001) compared to cells cultured on hTERT-MSC dECM and ^▲▲▲^(*p* value < 0.0005) compared to cells cultured on hFibroblast dECM.

**FIGURE 10:**
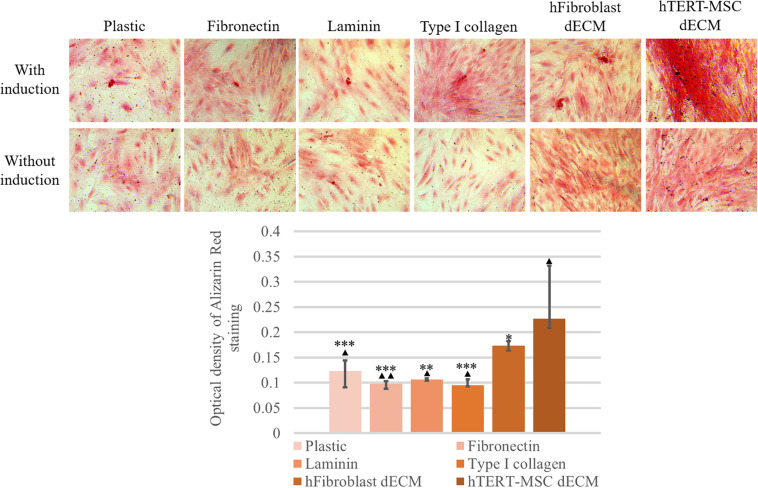
Induction of osteogenic differentiation of hMSCs cultured on plastic, plastic covered by fibronectin, or laminin, or type I collagen and dECM produced by human fibroblasts or MSCs for 4 days assessed by histological staining with Alizarin Red. Objective magnification, ×20. The dye was extracted, and Alizarin Red staining was quantified by measuring the optical density at 560 nm **(lower panel)** (*n* = 6). The quantitative data are represented as median (25%, 75%), ^∗^(*p* value < 0.05), ^∗∗^(*p* value < 0.005), ***(*p* value < 0.0005) compared to cells cultured on hTERT-MSC dECM and ^▲^(*p* value < 0.05), ^▲▲^(*p* value < 0.005) compared to cells cultured on hFibroblast dECM.

**FIGURE 11:**
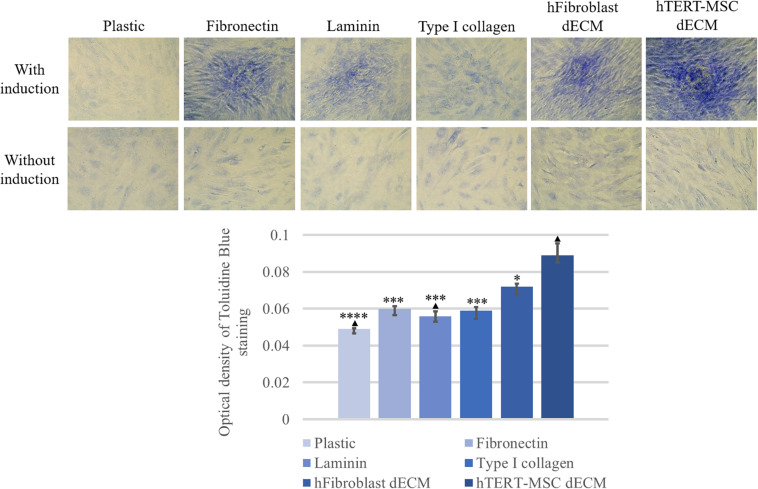
Induction of chondrogenic differentiation of hMSCs cultured on plastic, plastic covered by fibronectin, or laminin, or type I collagen and dECM produced by human fibroblasts or MSCs for 4 days assessed by histological staining with Toluidine Blue. Objective magnification, ×20. The dye was extracted, and Toluidine Blue staining was quantified by measuring the optical density at 608 nm **(lower panel)** (*n* = 6). The quantitative data are represented as median (25%, 75%), ^∗^(*p* value < 0.05), ^∗∗∗^(*p* value < 0.0005) compared to cells cultured on hTERT-MSC dECM and ^▲^(*p* value < 0.05) compared to cells cultured on hFibroblast dECM.

MSCs derived from adipose tissue represent heterogenic cell population with a number of multipotent cells capable to be induced to differentiate into adipocytes, osteocytes and chondrocytes. This property underlies some of the regenerative effects of MSCs *in vivo*. We tested the ability of dECM to modulate induced differentiation of these cells into the adipogenic and osteogenic lineages *in vitro* to evaluate a possible role of MSC-produced ECM in this process. hMSCs were cultured on dECM derived from hTERT-MSCs or fibroblasts, or on plastic covered with type I collagen, fibronectin or uncovered for 10 days under inductive conditions and then were stained with OilRed for adipocytes and Alizarin Red for osteocytes (Figures 9, 10). We observed that dECM derived from hTERT-MSCs significantly accelerated both adipogenic and osteogenic differentiation of hMSCs compared with the differentiation of cells cultured in different conditions. The difference was detectable even on the 4th day while the greatest differences were observed on the 10th day. At the same moment hMSC cultured on the plastic shown just the first signs of differentiation (Figures 8A–D, 9, 10). Differentiation was confirmed by real-time PCR. Although due to the high level of variability, mRNA level of marker genes for adipogenic (*PPAR*γ, *ADIPOQ*) and osteogenic (*BGLAP, RUNX2*) differentiation only tended to have higher level in cells cultured on dECM compared to plastic (Figures 8E–I). Importantly, we found that dECM effectively promoted differentiation of hMSC into adipogenic and osteogenic lineages within specific induction culture conditions at the very early stages (day 4 after induction of differentiation) compared not only to plastic, but also to plastic covered by selected ECM components (fibronectin, or type I collagen or laminin) presented in dECM (Figures 9–11). Interestingly, dECM produced by human fibroblasts and processed using the same protocol couldn’t enhanced hMSC differentiation like hTERT-MSCs-produced dECM, indicating cell-specific functionality of dECM.

### Attachment to dECM Modulates Specific Cell Signaling Pathways in hMSCs

**FIGURE 12:**
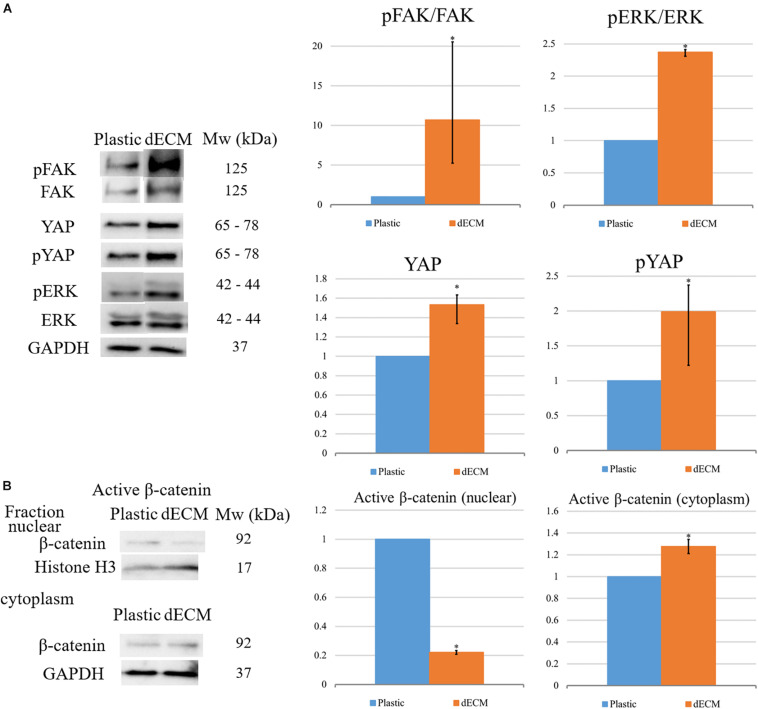
Evaluation of differentiation-related intracellular signaling pathways activation in hMSCs cultured on plastic or dECM in total lysate of samples for targets: **(A)** Evaluation of activation of FAK, ERK, pYAP and YAP. The quantitative data are represented as folds of protein expression in hMSCs cultured on dECM vs. hMSCs cultured on plastic, data are median (25%, 75%), *p < 0.05; **(B)** Analysis of distribution of active β-catenin between nucleus and cytoplasm. The quantitative data are represented as folds of protein expression in hMSCs cultured on dECM vs. hMSCs cultured on plastic, data are median (25%, 75%), ^∗^(*p* value < 0.05). Figure shows representative blots, *n* = 3.

To reveal the possible mechanisms of the observed dECM-mediated effects on hMSC differentiation, we analyzed the changes in the intercellular signaling pathways in hMSCs cultured on dECM. The outside-in signals from ECM are transferred due to the interaction of cell surface with chemical ECM components (mainly by integrins) as well as by the sensing of ECM stiffness, fiber orientation or other mechanical forces by cells. To check if hMSCs were able to form contacts with dECM via integrins and sense mechanical characteristics of their microenvironment, we analyzed the activation of key signaling pathways in hMSCs after the culture on plastic or dECM by immunoblotting (Figure 12). The phosphorylation level of focal adhesion kinase (FAK) was increased in hMSCs seeded on dECM compared to plastic which indicated the upregulation of integrin signaling due to binding of cell surface integrins to ECM proteins and confirmed the functionality of prepared dECM. Moreover, we observed the increase of downstream FAK target phosphorylation like ERK 1/2 (p42/p44) after attaching of hMSCs to dECM. Hippo effectors YAP/TAZ are known as on–off mechanosensing switches for ECM, so we examined pYAP and YAP expression in hMSCs and found the slight elevation of both protein forms in cells cultured on dECM. On the other hand, interplay between hMSCs differentiation and adhesion may be mediated by b-catenin signaling (Takada et al., 2009). We observed the reduction of active β-catenin level in nuclear fraction of hMSCs on dECM compared to cells cultured to plastic (Figure 11). This may be a result of increased recruitment of b-catenin to the forming cell–matrix contacts which are known to compete with nuclear factors for β-catenin (Brembeck et al., 2004).

### dECM Supports hMSC Differentiation Through Src-PI3K-AKT-Dependent Cell Signaling Pathway

**FIGURE 13:**
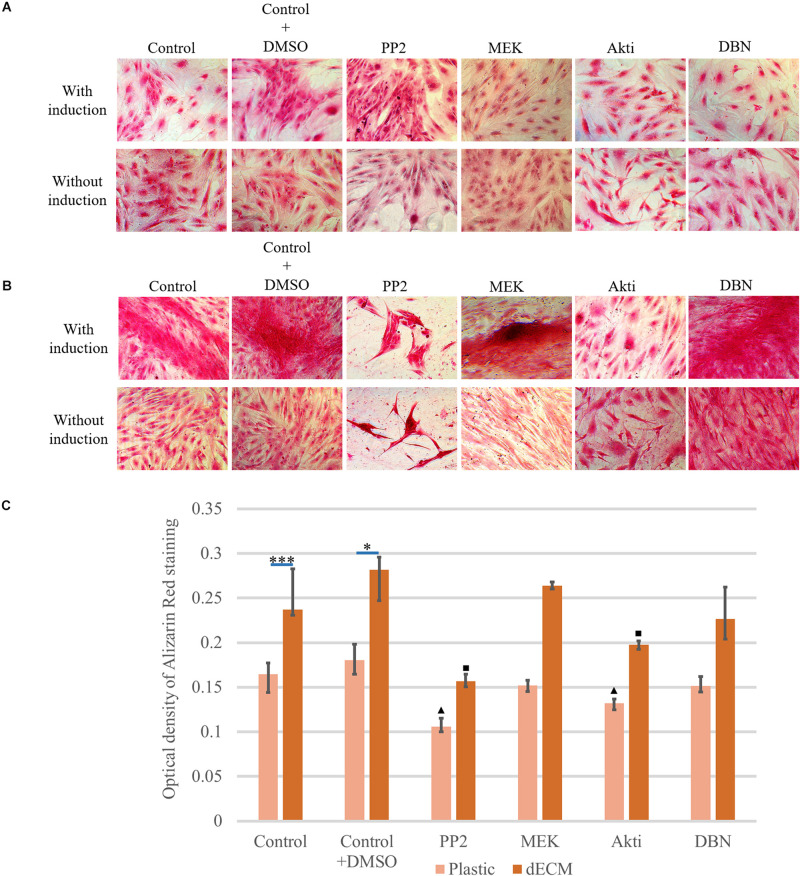
Inhibition study of induction mechanism of osteogenic differentiation with inhibitor treatment (PP2 as Src kinase inhibitor, MEK as ERK inhibitor, Akti VIII(Akti) as protein kinase B (Akt) inhibitor and dobutamine (DBN) as YAP inhibitor) of hMSCs cultured on plastic **(A)** or dECM produced by hTERT-MSCs **(B)** for 4 days assessed by histological staining with Alizarin Red. Objective magnification, ×20. The dye was extracted, and Alizarin Red staining was quantified by measuring the optical density at 560 nm **(lower panel C)** (*n* = 3). The quantitative data are represented as median (25%, 75%), ^∗^(*p* value < 0.05), ^∗∗∗^(*p* value < 0.0005), triangle and square signs represent the significant changes compared to the corresponding controls (*p* value < 0.05).

Despite the fact that our preliminary results demonstrated the classical activation of the ERK signaling cascade for cell interaction with ECM (Mecham, 2011), the Src-PI3K-AKT signaling pathway was revealed to be critical for rapid activation of differentiation on dECM (Figure 13Supplementary Figure S7). First, the cells that were cultured on dECM were extremely sensitive to the Src inhibitor. This was manifested in a strong change of the morphology of cells and in formation of spheroid-like structures. A similar process was observed during inhibition of FAK of ectomesenchymal multipotent stem cells when in cell culture dishes coated with poly-L-lysine (Beck et al., 2014). In the presence of Src inhibitor the ability of dECM to promote hMSC differentiation significantly decreased (Figure 13). At the same time, the addition of MEK and DBN inhibitors did not in have any effect on the phenomena we observed. As for the Acti inhibitor, it did not change the cell morphology, but it did inhibit the dECM-mediated stimulation of hMSC differentiation, which we observed earlier. PI3K/Akt signaling is a key pathway required for human MSC osteogenesis (Baker et al., 2015). These facts suggest that the Src-PI3K-AKT signaling pathway plays an important role in stimulating cell differentiation in the activated microenvironment.

### Interaction of hMSCs With dECM via Integrins May Modulate Cell Differentiation

**FIGURE 14:**
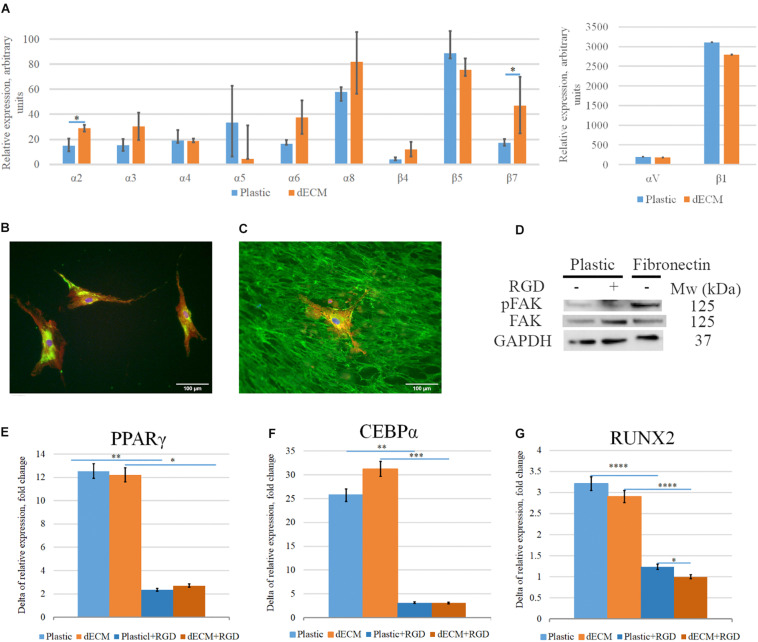
Impact of interaction of hMSCs with dECM via integrins into dECM-mediated hMSCs differentiation stimulation. **(A–C)** Expression of integrins in hMSCs cultured on plastic **(A,B)** or dECM **(A,C)** estimated by real-time PCR **(A)** including α2(*ITGA2*), α3(*ITGA3*), α4(*ITGA4*), α5(*ITGA5*), αV(*ITGAV*), α6(*ITGA6*), α8(*ITGA8*), β1(*ITGB1*), β4(*ITGB4*), β5(*ITGB5*), β7(*ITGB7*) and immunocytochemical staining for integrin β1 subunit **(B,C)**. **(D)** Activation of pFAK/FAK signaling pathway in hMSCs in suspension (1) on the presence of RGD peptide (2) or after 1-h attachment to fibronectin (3). **(E–G)** Relative fold change of hMSC differentiation induction (4 days) measured as increased expression of differentiation markers for adipogenic [PPARγ(*PPARG*) **(E)**, CEBPα(*CEBPA*) **(F)**], and osteogenic [*RUNX2* **(G)**] differentiation by real-time PCR normalized to housekeeping gene (RPLP0) in hMSCs cultured on plastic or dECM with or without blocking of integrin interaction via RGD sequence using RGD peptide. The quantitative data are represented as median (25%, 75%), ^∗^(*p* value < 0.05), ^∗∗^(*p* value < 0.005), ^∗∗∗^(*p* value < 0.0005), ^*⁣*⁣**^(*p* value < 0.0001).

Considering the significant activation of pFAK/FAK signaling pathway in hMSCs cultured on dECM we evaluated the impact of integrin-mediated interactions into the stimulation of hMSCs differentiation. We observed the expression of the wide range of integrin genes including α2, α3, α4, α5, αV, α6, α8, β1, β4, β5, β7 subunits in hMSCs (Figure 14A), although we didn’t find any significant differences between mRNA levels of these genes in hMSCs cultured on plastic vs dECM, except the expression of α2 and β7 subunits which was slightly higher in hMSC cultured on dECM. High expression level was observed for *ITGAV, ITGA8, ITGB1* and *ITGB5*, while we confirmed β1 subunit expression on hMSCs using immunohistochemical analysis (Figures 14B,C). Possible combinations of αVβ1, αVβ5, α8β1 probably play important role in interaction of heterogeneous hMSCs population with ECM components such as fibronectin and vitronectin which in most cases involves RGD sequence. To evaluate the impact of this type of cell-ECM interaction, we blocked it with RGD-peptide, which prevents binding of integrins to RGD-containing ECM proteins, during the induction of adipogenic or osteogenic differentiation. The optimal concentration of RGD peptide was selected based on the preliminary experiments when ability of RGD concentration to prevent adherence for 4 days. We also confirmed that addition of RGD peptide couldn’t activate pFAK/FAK signaling pathway in hMSCs by contrast with attachment to fibronectin (Figure 14D). Importantly, in the presence of RGD peptide the expression of the master-gene for adipogenic differentiation *PPAR*γ and *CEBP*α significantly inhibited on the 4^th^ day after the differentiation induction (Figures 14E,F). Correspondingly, the addition of RGD peptide during the induction of osteogenic differentiation significantly decreased mRNA level of *RUNX2*, which is considered as master-gene of this process and was markedly induced by induction medium (Figure 14G), while the level of *BGLAP* as well as *ADIPOQ* mRNA did not change (data not shown) in hMSC cultured on dECM during osteogenic and adipogenic differentiation, respectively.

## Discussion

The rapid development of cell-based technologies leads to the transition from artificial biomaterials to modulation of regenerative processes involving organism endogenous resources and mimicking the natural healing processes. One of the perspective approaches is a reconstitution of cellular microenvironment, or niche. Niche is a specialized cell microenvironment which refers to the extrinsic physical and functional factors that feedback to mediate cell behavior (Schofield, 1978). Only being in the specific niche a cell can retain its properties (Nimiritsky et al., 2018; Andreotti et al., 2019; Pastuła and Marcinkiewicz, 2019; Lee et al., 2020). Tissue damage leads to the disturbing of niches, which can be the reason why tissue or organ cannot recover completely. Many approaches are aimed to creating a biomaterial similar to the natural microenvironment (Donnelly et al., 2018; Hagbard et al., 2018; Thomas et al., 2018; Shrestha and Yoo, 2019). The difficulty lies in the selection of niche components that could be crucial for reconstruction of naïve microenvironment. Thus, ECM is an important niche component in regulation of cell behavior, including cell adhesion, proliferation and differentiation (Cramer and Badylak, 2019; Padhi and Nain, 2020). The effects of selected ECM components may depend on their molecular weight (Garantziotis and Savani, 2019; Wahart et al., 2019) and tissue specificity (Smith and Gerecht, 2018; Padhi and Nain, 2020).

The development and application of dECM matrices, prepared both by decellularization of tissues or whole organs and cell-based constructs, has grown rapidly in the fields of cell biology, tissue engineering and regenerative medicine in recent years (Hoshiba, 2017; Rana et al., 2017; Shakouri-Motlagh et al., 2017; Parmaksiz et al., 2020; Riis et al., 2020; Sart et al., 2020). The source of cells producing ECM is of great importance for realizing its functional role (Cramer and Badylak, 2019). Among other cell types MSCs represent a promising source of multipotent adult stem and progenitor cells for cell therapy and tissue engineering (Lukomska et al., 2019; Gomez-Salazar et al., 2020). They play an important role in maintenance of hematopoietic stem cell niche and are considered as a critical stem cell niche component in different tissues (Kfoury and Scadden, 2015). Accumulating evidence indicated that MSC regenerative effects are mostly mediated by their ability to produce a wide range of bioactive molecules such as growth factors (Chen et al., 2019; Dilogo and Fiolin, 2019; Seetharaman and Srivastava, 2019) and cytokines (Selich et al., 2019), extracellular vesicles (Keshtkar et al., 2018; Gowen et al., 2020) as well as ECM components (Kalinina et al., 2015; Ragelle et al., 2017; Rolandsson Enes et al., 2017; Devaud et al., 2018). Extracellular matrix production by MSCs is enhanced if cells are cultured as cell sheets which can be used as cell-derived matrices after decellularization. MSC-produced dECM has recently emerged as a promising substrate for the improved expansion of different cells (Chen et al., 2007; Lai et al., 2010; Ng et al., 2014; Wu et al., 2016). It was shown that MSCs cultured on these surfaces exhibited improved proliferation capacity, maintenance of phenotype, and increased differentiation potential (Shakouri-Motlagh et al., 2017; Sart et al., 2020). However, the mechanisms of these effects remained to be poor understood.

In the current study we have tested several agents for decellularization of hTERT-MSCs cell sheets (detergents, enzymes, apoptosis inductors). It should be noted that cellular sheets as structures do not have such a potent ECM as in the formed organs, it is necessary to carefully select the agents for decellularization to preserve the functional biologically active substances and to form the more correct model of cell microenvironment *in vitro* (Gilpin and Yang, 2017). Our experimental findings allowed to select the optimized protocol of hTERT-MSCs cell sheet decellularization using the combination of brief incubation with CHAPS and the following DNAse I treatment with high efficiency of DNA removal and preservation of ECM proteins, including fibronectin, type I collagen and laminin. We did not reveal the presence of fibrillar proteins in native cell sheets or in dECM by two-photon microscopy. However, according to other authors, fibrillar proteins could be found in the cell layers produced by the primary isolated bone marrow and adipose tissue-derived MSCs (Marinkovic et al., 2016) which might be explained by methodological differences.

Residual DNA in dECM may be immunogenic for cell culture or cause immune response when injected *in vivo*. However, in our study prepared dECM did not stimulate THP-1 macrophage differentiation which was evaluated by cell attachment to the substrate and specific cytokine profile.

The maintenance of stemness is one of the crucial functions of stem cell niche. As MSCs are involved in niche functioning *in vivo* it is important to understand if they perform this particularly via production and organization of ECM. We tested this hypothesis by analyzing the expression of pluripotency genes (*NANOG, OCT4, SOX2*) in hMSCs and colonies formed by multipotent cells within heterogeneous population of hMSCs cultured on plastic or dECM in CFU assay. Interestingly, we revealed that not the number of colonies, but their average size increased if hMSCs were cultured on dECM, and these findings were confirmed by the data obtained by others (Rao Pattabhi et al., 2014). Correspondingly, there were no substantial differences between hMSCs cultured on plastic or dECM in pluripotency gene expression, although *SOX2* expression was slightly increased in hMSCs cultured on dECM. Thus, dECM may not improve the number of multipotent cells, but increased significantly clonal expansion of progenitors, and these findings were confirmed by the data obtained by others (Rakian et al., 2015; Ji et al., 2018). We also observed that the proliferative rate of total hMSCs population slightly increased on dECM comparing to plastic, which can be explained by selective stimulation of proliferation of multipotent progenitor cells wherein not the proliferation of differentiated cells.

The development of complex tissues, including various cell types and their spatial organization, depends on a consistent series of decisions about cell fate, which begins with the fertilization of a single cell. Although growth factors are usually regarded as the major extracellular ligands that control these decisions, ECM and intercellular adhesion provide equally important instructions for controlling gene expression and the fate of all cells. Integrins play central role to all these aspects of determining the fate of a cell. They transmit signals into the cell via adhesion complexes, which include several types of specialized cell interaction sites with ECM, including focal complexes, focal adhesions, fibrillar adhesions, and podosomes (Zaidel-Bar et al., 2007). Moreover, they recruit both adapters (such as paxillin, p130Cas, integrin-linked kinase, and guanine nucleotide metabolism factors), as well as enzymes (like protein kinases FAK, Src and Jnk, Rho-family GTPases and lipid kinases) that trigger signaling pathways controlling decisions about cell fate, such as survival, growth, migration, and differentiation (Giancotti and Tarone, 2003; Lo, 2006).

Cell differentiation is a result of certain combinations of transcription factor activity that cause cell type-specific gene expression patterns. Specific inductors can act alone or in complex with integrins as it was described for oligodendrocyte differentiation (Baron et al., 2005). According to our data, hMSCs plated on dECM turn on the integrin-mediated signaling via phosphorylation of FAK and downstream ERK signaling. This type of signals could be considered as pro-survival, pro-mitogenic and contributing to cell differentiation. We observed that hMSCs demonstrated rapid differentiation rate into three different lineages cultured on dECM vs plastic; moreover, this effect exhibited at the very early stages after induction and unlikely related to the differences in stiffness as it was not reproduced for hMSCs cultured on plastic covered by selected single ECM proteins (fibronectin, laminin or type I collagen) and dECM produced by fibroblasts in cell sheets. Using RGD peptide as an inhibitor of interactions between cell integrins and some key ECM proteins, we demonstrated that integrin-mediated signaling significantly contributed to the ability of dECM to support hMSC differentiation. This phenomenon may be partly mediated by the interaction of cells with fibronectin fibers. Soluble fibronectin has been shown to stimulate integrin signaling of cells. However, the presence of a filamentous structure with specific binding sites for ECM components can cause a synergistic effect of integrin signaling and growth factor signaling (Donnelly et al., 2018). When seeded on dECM, hMSCs maintained their phenotype, although in many cell types ERK signaling was induced directly by integrin adhesion (Velling et al., 2008). Probably selective switch-on of integrin-pFAK-pERK signaling in multipotent cells depends on different repertoire of integrins on cell surface comparing to stromal cell fraction. According to other author’s data, stem cells may represent wide spectrum of different integrin subunits which participate in maintenance of stemness (Saller et al., 2012). Furthermore, total MSCs population exhibit a lot of integrins on cell surface (Prowse et al., 2010), which correlates with our data on integrin expression by hMSCs. Thus, a multipotent cell may express unique integrins which mediate dECM binding and ERK phosphorylation which lead to increased clonal expansion.

Additionally, we observed an important role of Src-PI3K-AKT signaling pathway in stimulation of MSC differentiation by dECM. Inhibition of Src protein kinase dramatically changed morphology of cells plated on dECM and led to formation of spheroid-like structures, as well as resulted in decreased differentiation potential. We assume that this results from the loss of contact of cells with ECM, which leads to increased formation of intercellular contacts. However, selective inhibition of Akt did not affect cell morphology but only mitigated the pro-differentiation effect of dECM, suggesting that Akt acted downstream and affected only processes of differentiation but not cell–matrix interactions.

The interaction between dECM and cells also modulated the activity of canonical Wnt/b-catenin signaling pathway in hMSCs. b-catenin, the main effector of this signaling, exists in superposition of two conditions – on the one hand, connecting E-cadherin and a-catenin in cell adhesion complex (Drees et al., 2005), on the other, it can transfer to the nucleus and act as a transcriptional activator in conjunction with its partners LEF/TCF (Behrens et al., 1996). In our experiments we observed decrease of active b-catenin content in nuclear fraction of hMSCs cultured on dECM. This phenomenon may be a result of increased recruitment of b-catenin to the forming cell–matrix contacts, which are known to compete with nuclear factors for β-catenin (Brembeck et al., 2004).

Importantly, there was no specific cell commitment to adipogenic, osteogenic or chondrogenic lineages for hMSCs as could be expected from some previously published data (Marinkovic et al., 2016). We consider this non-specific effect due to the transient activation of ERK signaling cascade in multipotent progenitor cells on dECM, which is particularly necessary for the initial stage of adipogenic differentiation (Tyurin-Kuzmin et al., 2020). Our hypothesis is also confirmed by the slight elevation of both pYAP and YAP expression in hMSCs cultured on dECM. YAP is an important effector protein in the Hippo signaling pathway that acts as a transcriptional regulator of several transcription factors activity, including RUNX, and provides the mechanosensing switches for ECM. Increased YAP expression is essential for satellite cell activation under homeostasis in skeletal muscles, whereas pYAP gradually elevates along with the expansion and differentiation of muscle progenitor cells (Fischer et al., 2016; Eliazer et al., 2019). Other examples include progenitors in the intestinal crypt, neural progenitor cells in the neural tube and epidermal stem cells, where YAP activity promotes proliferation and blocks differentiation (Camargo et al., 2007; Cao et al., 2008; Watt et al., 2010; Schlegelmilch et al., 2011). Pancreatic exocrine cells are also converted to progenitors by transient YAP expression (Panciera et al., 2016). Recently it was shown that YAP is also crucial for modulating human MSC differentiation to adipocytes and osteoblasts (Lorthongpanich et al., 2019).

Taken together, based on all revealed changes in hMSCs cultured on dECM we suppose that specific MSC-produced ECM-mediated signaling leads to the proliferation of multipotent cells and generation of progenitors, increasing the number of cells able to be quickly and efficiently induced into a differentiation process. It is worthy to note that the cell embedded in the thickness of ECM should be able to recognize the specific signals from the outside. The diverse responses to the extrinsic stimuli may be due to the dose-dependent activities of signaling factors, or to intrinsic differences in the response of cells to a given signal—a phenomenon called differential cellular competence (Kiecker et al., 2016). We found that dECM simultaneously smoothed out the basal expression level of differentiation transcription factors in hMSCs, supported proliferation of progenitor cells and prepared the intracellular pathways for the rapid response phase. Thus, we assume that ECM secreted by MSCs could convert target cells to the competent state facilitating their responses to the specific differentiation stimuli. Whether these effects are distinctive for cultured MSCs or realizing also *in vivo*, need further investigations.

## Conclusion

ECM represents a dynamic structure, actively remodeling throughout life. Producing of ECM components and creating of regulatory framework for the rest of cell types is one of the crucial functions of MSCs. Moreover, produced ECM does not only provide a mechanical support, but plays a decisive role in formation of cell microenvironment and regulation of cell fate, in particular within stem cell niches. Our approach of MSC cell sheet decellularization allowed us to trace the role of MSC-produced ECM in multipotent cell proliferation and differentiation. The obtained data suggest that ECM can stimulate these processes via binding of integrins with RGD sequences well-conserved in dECM samples. Thus, MSC-produced dECM largely reconstitutes composition and structure of the native matrix, so it may mimic stem cell niche components *in vitro* and maintain multipotent progenitor cells to insure their effective response to the external differentiating stimuli. MSC cell sheets and obtaining dECM from such multicellular constructions represent the promising *in vitro* models for the establishing of ECM contribution into the organization of specific cell microenvironment in various conditions. Our results also can be used for the developing of novel biomedical cell-free products based on dECM for regenerative medicine.

## Data Availability Statement

All datasets presented in this study are included in the article/Supplementary Material.

## Ethics Statement

The studies involving human participants were reviewed and approved by Ethics Committee of Lomonosov Moscow State University, IRB00010587. The patients/participants provided their written informed consent to participate in this study.

## Author Contributions

EN, OG, and AE designed the study. EN performed all the experiments, except those specified below. SR provided with DIC microscopy and study of SHG. OG and RE helped sample preparation for IHC of ECM components and fluorescence microscopy. PN and OG helped with THP-1 and ELISA experiments. PN estimated the amount of ATP in samples. KK and NB helped with RT PCR for differentiation experiments. KK, MK, and IM provided western-blot. EN, OG, KK, NO, and AE provided intellectual input to the manuscript. EN, OG, KK, and AE wrote the manuscript. All authors contributed for the development of this article.

## Conflict of Interest

The authors declare that the research was conducted in the absence of any commercial or financial relationships that could be construed as a potential conflict of interest.

## Footnotes

1 http://www.ncbi.nlm.nih.gov/tools/primer-blast/

## Supplementary Material

FIGURE S1 Apoptosis of hTERT-MSCs caused by rotenone. The flow cytometry data obtained for unlabeled cells **(A)** and after labeling of cells with Annexin V and 7-AAD before **(B)** and after the incubation with rotenone (500 nM) for 24 h **(C)**. Effectiveness of apoptosis induction in hTERT-MSC cell sheet using rotenone is shown on diagram **(D)**.

FIGURE S2 Expression of ECM components such as fibronectin **(A–E)**, type I collagen **(F–J)**, laminin **(K–O)** in hTERT-MSC cell sheets **(A,F,K)** and dECM prepared by different decellularization methods: CHAPS 10’ with DNAse I **(B,G,L)**, rotenone pretreatment with the following decellularization by CHAPS and DNAse I **(C,H,M)**, sodium deoxycholate with DNAse I **(D,I,N)**, rotenone pretreatment with the following decellularization by sodium deoxycholate and DNAse I **(I,J,O)** evaluated by immunohistochemistry without permeabilization (objective magnification, x40). The IgG control is presented for each sample in the corresponding column **(P–T)**.

FIGURE S3 Scanning electron microscopy of MSC cell sheets **(A,F)** and dECM prepared by different decellularization methods: CHAPS with DNAse I **(B,G)**, rotenone pretreatment with the following decellularization by CHAPS and DNAse I **(C,H)**, sodium deoxycholate with DNAse I **(D,I)**, rotenone pretreatment with the following decellularization by sodium deoxycholate and DNAse I **(I,J)**. Magnification: x1000 **(A–E)**, x4000 **(F–J)**.

FIGURE S4 Isotype IgG control immunocytochemical staining without permeabilization of MSC cell sheets **(A)** and dECM **(B)** for Figure 3.

FIGURE S5 Isotype IgG control immunocytochemical staining without permeabilization of hMSC on dECM **(A)** and TCP **(B)** for Figure 12.

FIGURE S6 Proliferation activity of activated monocytes/macrophages (THP-1) cultured on plastic and dECM. **(A)** representative microphotographs of THP-1 cultured on plastic and dECM with or without PMA treatment in the beginning and for 4 days (phase contrast, objective magnification – ×10). Additional cytokine profile of monocytes cultured on plastic and dECM. Level of IL-8 **(B)** and IL-10 **(C)** secreted by monocytes/macrophages with or without PMA treatment measured by ELISA are presented. The quantitative data are represented as median (25%, 75%).

FIGURE S7 Inhibitor analysis of major signaling pathways in hMSC cultivated on plastic or dECM. DBN-dobutamine, Mek – MEK inhibitor PD 98059, PP2 – Src inhibitor PP2, Akti – Akt1 and Akt2 inhibitor Akti-1/2. Figure shows representative blots, n = 3.
