# Supplementary figures and images for: Mesenchymal Stromal Cell-Produced Components of Extracellular Matrix Potentiate Multipotent Stem Cell Response to Differentiation Stimuli
Source: Front Cell Dev Biol. 2020 Sep 22;8:555378. doi: 10.3389/fcell.2020.555378 (PMC7536557; doi:10.3389/fcell.2020.555378)

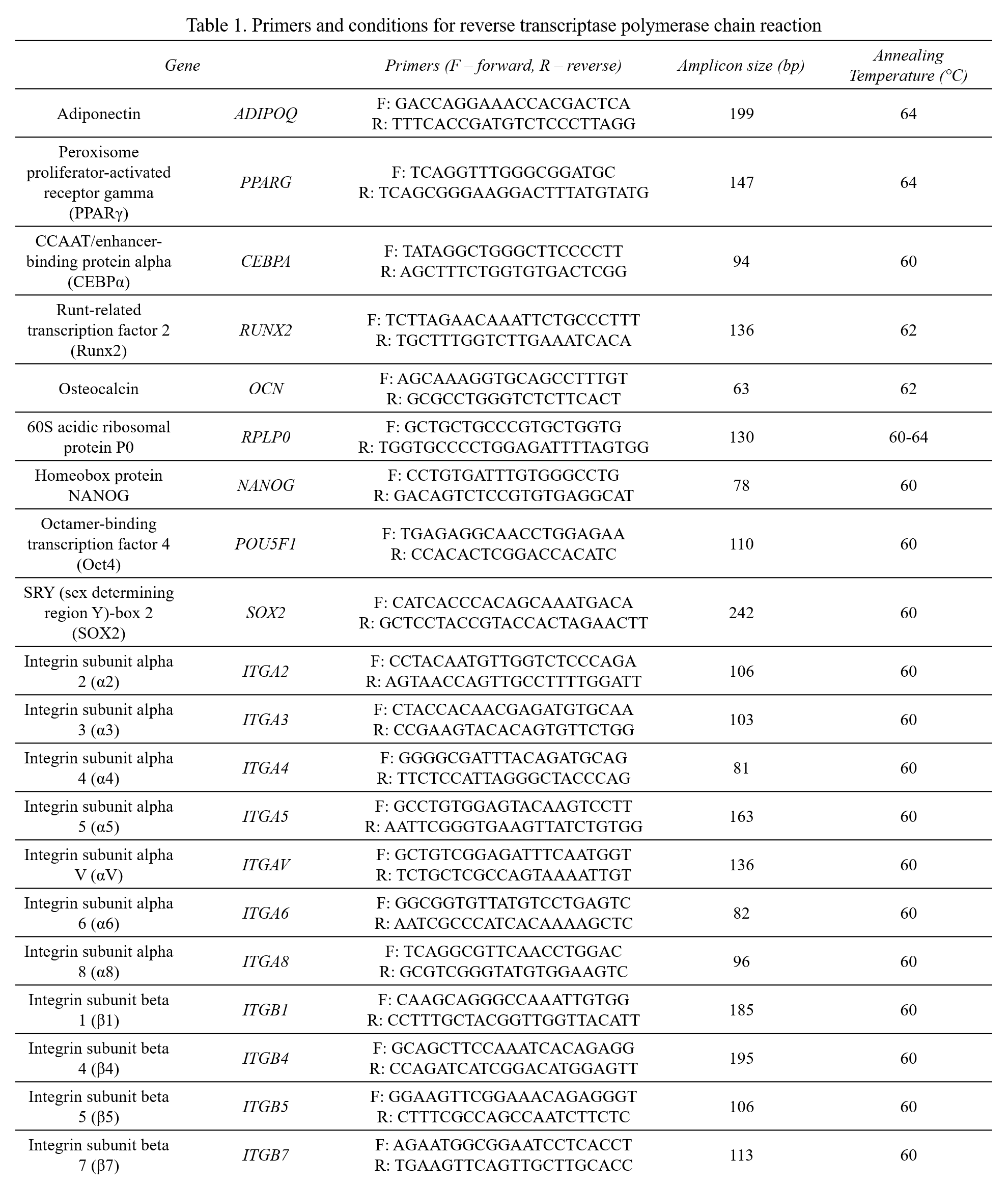

Supplement: FIGURE S1 — Apoptosis of hTERT-MSCs caused by rotenone. The flow cytometry data obtained for unlabeled cells (A) and after labeling of cells with Annexin V and 7-AAD before (B) and after the incubation with rotenone (500 nM) for 24 h (C). Effectiveness of apoptosis induction in hTERT-MSC cell sheet using rotenone is shown on diagram (D). [file Image_1_v1.TIF]

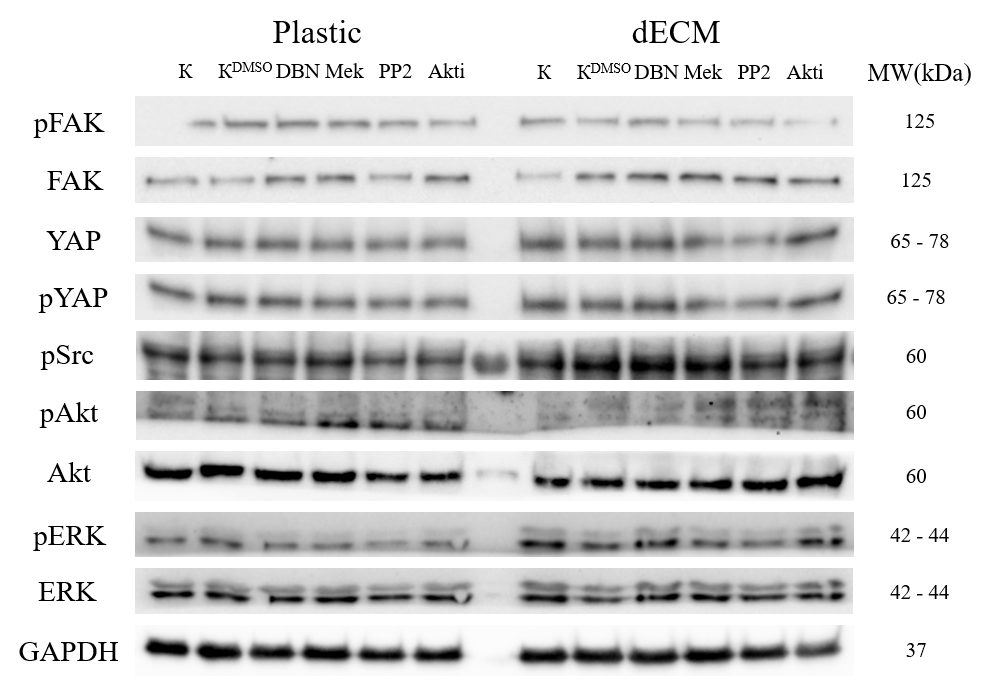

Supplement: Supplementary file 8 [file Image_8_v1.TIF]
